# Modeling fragment counts improves single-cell ATAC-seq analysis

**DOI:** 10.1038/s41592-023-02112-6

**Published:** 2023-12-04

**Authors:** Laura D. Martens, David S. Fischer, Vicente A. Yépez, Fabian J. Theis, Julien Gagneur

**Affiliations:** 1 School of Computation, Information and Technology, Technical University of Munich, Garching, Germany; 2 Computational Health Center, Helmholtz Center Munich, Neuherberg, Germany; 3 Helmholtz Association, Munich School for Data Science (MUDS), Munich, Germany; 4 TUM School of Life Sciences Weihenstephan, Technical University of Munich, Freising, Germany; 5 Institute of Human Genetics, School of Medicine, Technical University of Munich, Munich, Germany

**Keywords:** Machine learning, Computational models

## Abstract

Single-cell ATAC sequencing coverage in regulatory regions is typically binarized as an indicator of open chromatin. Here we show that binarization is an unnecessary step that neither improves goodness of fit, clustering, cell type identification nor batch integration. Fragment counts, but not read counts, should instead be modeled, which preserves quantitative regulatory information. These results have immediate implications for single-cell ATAC sequencing analysis.

## Abstract (web summary)

This paper proposes quantitative modeling of single-cell ATAC-seq data, which improves various downstream analyses.

## Main

**Fig. 1:**
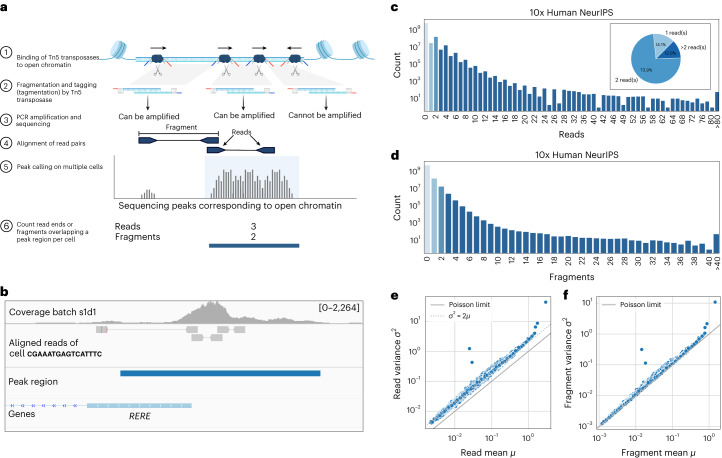
scATAC-seq data are quantitative and fragments, rather than reads, should be counted. **a**, Illustrated is the scATAC-seq protocol and count aggregation strategy. Tn5 transposases insert into open chromatin regions, cut the DNA and attach sequencing adaptors (blue and red). Two Tn5 insertions create one fragment with adaptors. The orientation of the insertion is important as only fragments flanked with two distinct barcodes can be captured and amplified. Fragments are sequenced paired-end and aligned to the genome. scATAC-seq peak calling is performed using reads from multiple cells. Once peak regions are identified, reads (deduplicated fragment ends) or fragments overlapping the peak region are counted for each cell separately. **b**, Genome viewer snapshot of one peak region in the NeurIPS dataset at the promoter of the human gene *RERE* showing multiple insertions in a single cell. The tracks show, from top to bottom, the coverage of one batch used for peak calling, the aligned read pairs of a single cell, the peak region and genome annotation. The peak region overlaps with five reads and three fragments. **c**, Read count distribution on the entire NeurIPS dataset. The striking odd/even pattern in read count distribution reflects that reads come in pairs and suggests that fragment counts, rather than reads, should be modeled. Pie chart showing the percentage of all non-zero peaks with one, two or more than two reads (inset). **d**, Distribution of the approximated fragment count does not show an even/odd pattern. **e**, Variance of read counts across cells against mean read counts. Each dot represents one peak region. When fragment ends (reads) are counted, the variance of read counts is about twice the mean (gray dotted line), which is not consistent with a Poisson distribution (solid gray line). **f**, Same as **e**, but for fragment counts. The variance of fragment counts is approximately equal to the fragment count mean, consistent with a Poisson distribution (solid gray line).

Single-cell assay for transposase-accessible chromatin using sequencing (scATAC-seq)^1^ is a major method employed to study chromatin regulation^2^. It employs Tn5 transposase to insert sequencing adaptors into accessible genome regions, resulting in reads representing Tn5 insertions in individual cells^1^ (Fig. 1a,b). When analyzing scATAC-seq data, open chromatin regions are generally identified on the pooled data as peaks, which are genomic regions with a significant excess of reads compared to the background^1,3,4^. Alternative approaches define the feature set as genomic windows or bins^5,6^ (Supplementary Table 1). Subsequently, the reads overlapping each feature are counted for each cell, yielding a typically very sparse matrix with less than 10% non-zero counts^7^.

Machine-learning modeling of scATAC-seq data supports investigations of single-cell genome regulation, including identification of cell types, differentially accessible regions and transcription factor activity inference. The loss function and data representation are crucial determinants of a model’s predictive power. Many methods default to binarizing the count matrix due to overall data sparsity and the conceptualization of chromatin accessibility as a binary state^5–10^ (Supplementary Table 1). While some approaches handle the data quantitatively^3,11,12^, there exists no systematic evaluation of the impact of binarization.

**Extended Data Fig. 1:**
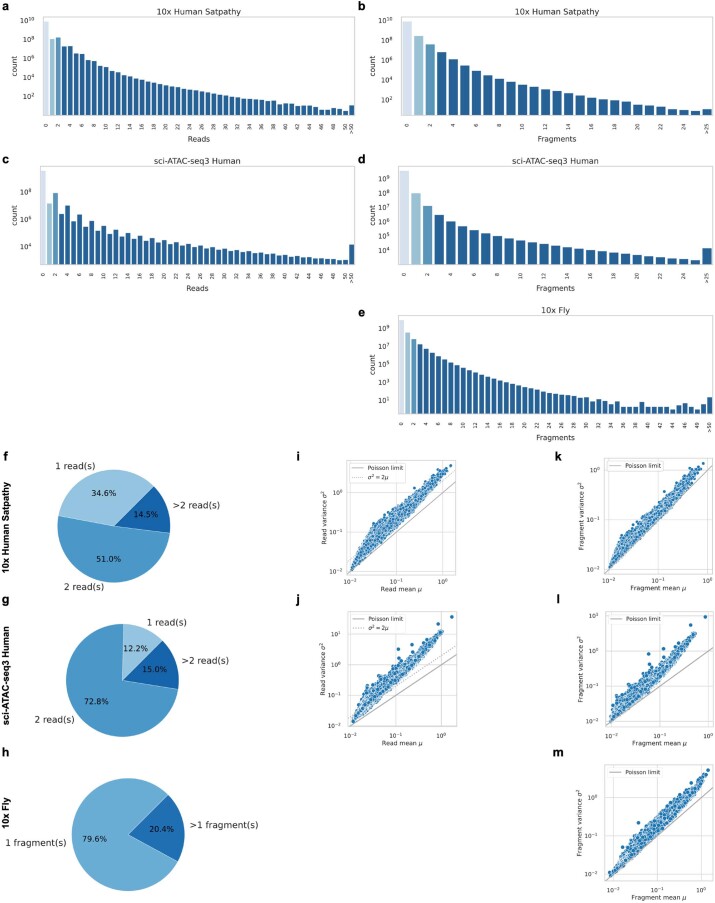
Comparison of read and fragment counts. **a, b)** Read count (a) and fragment count (b) distribution on the Satpathy dataset^14^. **c, d)** Read count (c) and fragment count (d) distribution of the sci-ATAC-seq3 dataset^16^. Plotted is a 10% random subset as the dataset consists of ~700 K cells. **e)** Fragment count distribution on the fly dataset^15^. CellRanger ATAC read counts were unavailable for this dataset as we generated fragment counts directly with Signac. **f, g)** Pie chart showing the percentage of all non-zero peaks with 1, 2, or more than 2 reads for the Satpathy dataset (f), sciATAC-seq3 dataset (10% random subset) (g). **h)** Pie chart with the percentage of all non-zero peaks with one or more than one fragment for the fly dataset (read counts are not available for this dataset). **i, j)** Variance of read counts across cells against mean read counts for the Satpathy dataset (i) and sciATAC-seq3 dataset (j). Each dot represents one peak region. When fragment ends (reads) are counted, the variance of read counts is around twice the mean (gray dotted line), which is not consistent with a Poisson distribution (solid gray line). **k, l, m)** Same as (i, j), but for fragment counts.

Here, we compare binarization versus count-based modeling on scATAC-seq data modeling tasks and assess the quality of the learnt latent space using multiple downstream evaluations. We based our analysis on four publicly available datasets representing different protocols, species and tissues^13–16^ (Supplementary Table 1; Methods). First, we considered the proportion of peaks above the typical binarization threshold of one read. Across all datasets, over 65% of non-zero peaks had more than one read count (Fig. 1c and Extended Data Fig. 1). In the NeurIPS dataset, for instance, 74% of non-zero peaks had counts of two, with 12% having even higher counts. We furthermore saw a fivefold increase in peaks with even compared to odd counts (Fig. 1c). This pattern can be explained as an artifact of the count aggregation strategy used in the 10x Genomics CellRanger ATAC pipeline^4^, which counts reads (deduplicated fragment ends) instead of fragments (Fig. 1a). As scATAC-seq generates paired-end reads, even counts are predominant, whereas odd counts only occur when one read pair falls outside the peak region (Fig. 1a,b). In contrast, fragment counts showed a regular monotonic decay (Fig. 1d and Extended Data Fig. 1; Methods). Many methods rely on the read count matrices generated by the 10x pipeline or adopt the same counting strategy^3,5–10,17^ (Supplementary Table 1); however, no benchmark has compared the read and fragment count strategies.

The alternating pattern of odd and even read counts does not align with standard statistical count distributions, such as the Poisson. We found that the variance of read counts for each region across cells was approximately twice the mean (Fig. 1e and Extended Data Fig. 1), violating the Poisson assumption of equal mean and variance. In contrast, the mean-variance relationship of fragment counts was broadly consistent with a Poisson distribution across the four datasets (Fig. 1f and Extended Data Fig. 1).

Altogether, these results have two implications. First, scATAC-data carries information beyond binary accessibility. Second, fragment counts, but not read counts, can be more suitably modeled with the Poisson distribution.

**Extended Data Fig. 2:**
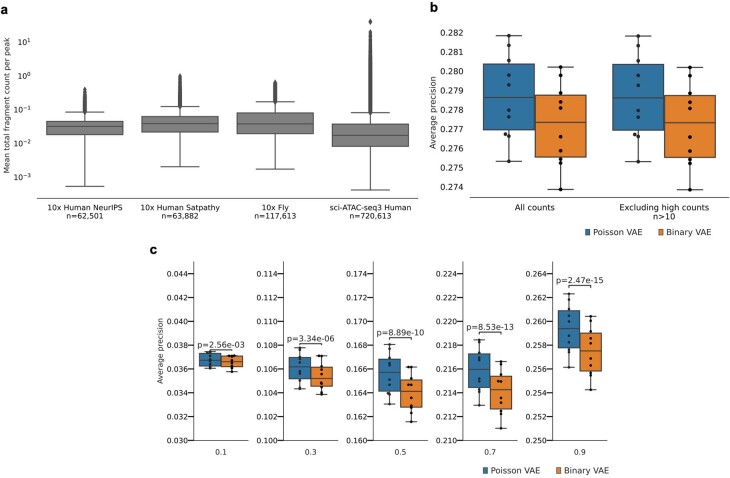
Fragment count distribution and performance evaluation with excluded high counts and downsampled data. **a)** Average fragment count distribution per peak for all four datasets. The sci-ATAC-seq3 dataset is 50% sparser than the 10x datasets. **b)** Average precision of the Poisson VAE and the Binary VAE model on the NeurIPS^13^ dataset for all cell-peaks and only the subset of cell-peaks with less than ten counts. **c)** Average precision for the Poisson VAE and Binary VAE model at different downsampling thresholds. *P* values were computed using the two-sided paired t-test. In boxplots, the central line denotes the median, boxes represent the interquartile range (IQR), and whiskers show the distribution except for outliers. Outliers are all points outside 1.5 times the IQR.

To assess how modeling fragment counts, rather than binarized signals, affects latent space learning, we adapted the PeakVI model, a state-of-the-art variational autoencoder (VAE) for scATAC-data^9^. Originally designed for binarized data, PeakVI learns the probability that a peak in each cell is accessible, while accounting for cell-specific effects and region biases through learnt factors. We modified PeakVI’s last layer to instead model Poisson-distributed fragment counts (Poisson VAE; Methods). As the total number of fragments per cell varies drastically across cells (Extended Data Fig. 2a), we incorporated the total fragment count as a precomputed offset in the loss instead of learning a cell-specific factor. Similarly, we tested the effect of including the precomputed offset in the binary case (Binary VAE; Methods).

**Fig. 2:**
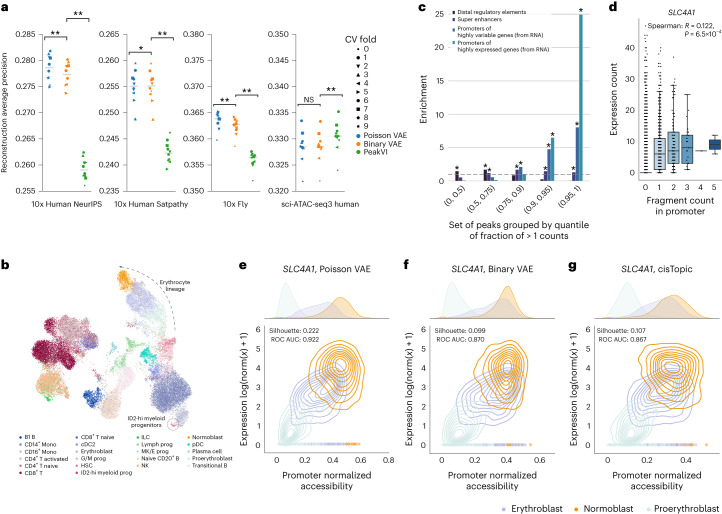
Binarizing scATAC-seq data is unnecessary and hides quantitative information. **a**, Comparison of the Poisson VAE, Binary VAE and PeakVI models on reconstructing the binarized cell-peak matrix of the NeurIPS, the Satpathy, the Fly and the sci-ATAC-seq3 datasets for ten cross-validation (CV) runs. Poisson VAE and Binary VAE use the observed total fragment count. The horizontal line denotes the median. *P* values were computed using a two-sided paired Wilcoxon test and Benjamini–Hochberg corrected. **P = 0.0019, *P = 0.0195, NS, not significant, *P* = 0.0695. **b**, Uniform Manifold Approximation and Projection (UMAP) of the integrated latent space of all NeurIPS batches, colored by cell type for the Poisson VAE model. The isolated label ID2-hi myeloid progenitors and the erythrocyte lineage are annotated. UMAPs for all other methods and datasets are in Extended Data Figs. 5–8. **c**, Enrichment (odds ratio, one-sided Fisher exact test) of distal regulatory elements, super-enhancers in bone marrow, promoters of highly expressed genes and promoters of highly variable genes in the scATAC-seq peaks of the NeurIPS dataset. Peaks are sorted by the fraction of counts above the binarization threshold and grouped according to different quantiles. *P < 0.0001. **d**, Correlation of expression of the *SLC4A1* gene and fragment counts in its promoter. The two-sided Spearman correlation analysis was computed on cells with at least one fragment count in the promoter (*n* = 775). The *P* values were adjusted for multiple testing using the Benjamini–Hochberg correction. We restricted the plot to cells of similar total fragment count (0.25–0.75 quantile) to not capture effects driven by total fragment count. **e**–**g**, log-normalized gene expression over normalized accessibility of the *SLC4A1* gene for the Poisson VAE (**e**), Binary VAE model (**f**) and cisTopic model (**g**). Cell type separation is measured with the silhouette width and area under the receiver operating characteristic (ROC) curve and is better with the Poisson VAE model. In all boxplots, the central line denotes the median, boxes represent the interquartile range (IQR) and whiskers show the distribution except for outliers. Outliers are all points outside 1.5 × IQR. AUC, area under the curve. B, B cell; T, T cell; Mono, Monocyte; prog, progenitor; HSC, Hematopoietic stem cell; ILC, Innate lymphoid cell; Lymph, Lymphoid; MK/E, Megakaryocyte and Erythrocyte; G/M, Granulocyte and Myeloid; NK, Natural Killer cell; cDC2, Classical dendritic celltype 2; pDCs, Plasmacytoid dencritic cells.

We first evaluated model performance across the four datasets by benchmarking them on predicting the presence of at least one read, the standard binarization threshold. For binary models, we used the predicted probability of a region being open, while for quantitative models, we converted predictions into the probability of having a count exceeding zero (Methods). There was no benefit from using binarized data in the 10x datasets as Poisson VAE significantly outperformed PeakVI and Binary VAE in reconstructing binarized counts (Fig. 2a). Notably, substantial performance gain was achieved by controlling for the observed rather than predicted total fragment counts as the binary model (Binary VAE) also showed significantly better reconstruction than PeakVI. We further tested that the performance improvement was not a result of disproportionately giving more weight to regions with high counts (Extended Data Fig. 2b). In contrast, the sparser sci-ATAC-seq3 dataset (median peak fragment count 0.036 versus 0.017 in the 10x datasets; Extended Data Fig. 2a and Supplementary Table 1), did not benefit from using quantitative information or the observed total fragment count. Downsampling of the NeurIPS dataset confirmed that the advantages of the quantitative model increased with a higher total fragment count (Extended Data Fig. 2c).

**Extended Data Fig. 3:**
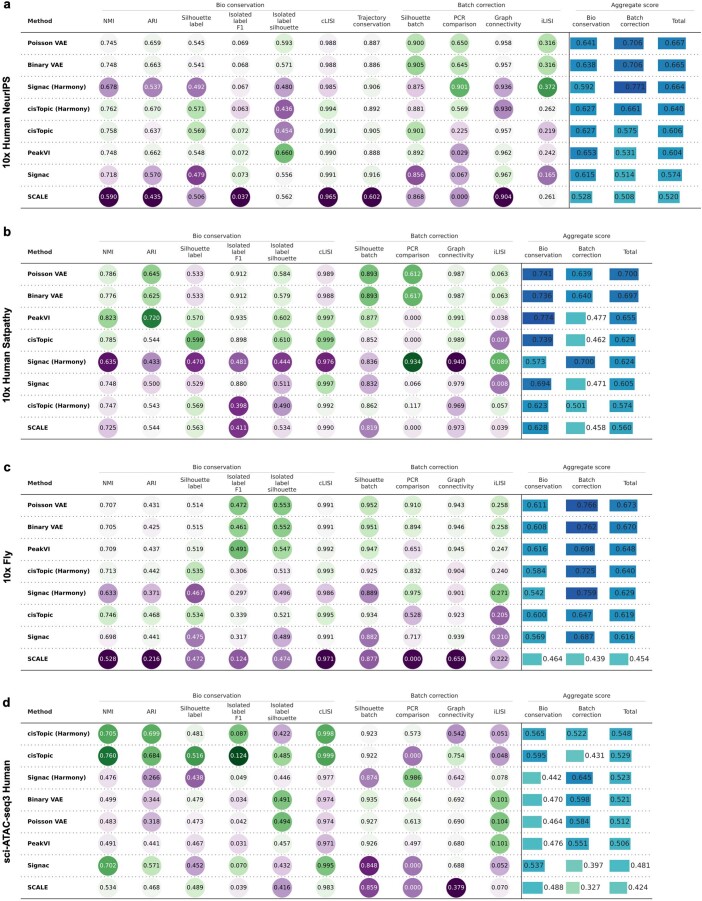
Full integration metrics per dataset. Comparison of integration accuracy for Poisson VAE, Binary VAE, PeakVI^9^, Signac^3^ using LSI, cisTopic^8^ using LDA and SCALE^10^ on (**a**) the NeurIPS, (**b**) the Satpathy (**c**) the fly and (**d**) the sci-ATAC-seq3 datasets. For cisTopic and Signac, additional batch correction was performed using Harmony^28^. Metrics are categorized into batch correction and bioconservation categories. Reported is the mean over ten cross-validation runs. Overall scores were computed using a 40:60-weighted mean of batch correction and bioconservation scores.

**Extended Data Fig. 4:**
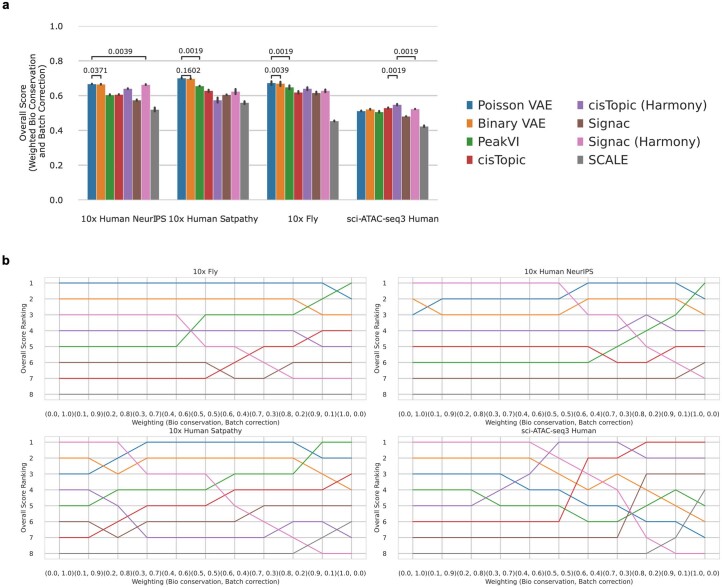
Overall score of integration including different weightings of bioconservation and batch correction. **a)** Comparison of integration accuracy for embeddings generated with Poisson VAE, Binary VAE, PeakVI, Signac, cisTopic, and SCALE on the four datasets. For cisTopic and Signac, additional batch correction was performed using Harmony. Overall integration accuracy scores were computed using a 40:60-weighted mean of batch correction and bioconservation scores. *P* values were computed using the two-sided paired Wilcoxon test; Benjamini–Hochberg corrected. Error bars represent the 95% confidence interval over ten cross-validation runs. **b)** Overall score computed from different bioconservation and batch correction weightings.

**Extended Data Fig. 5:**
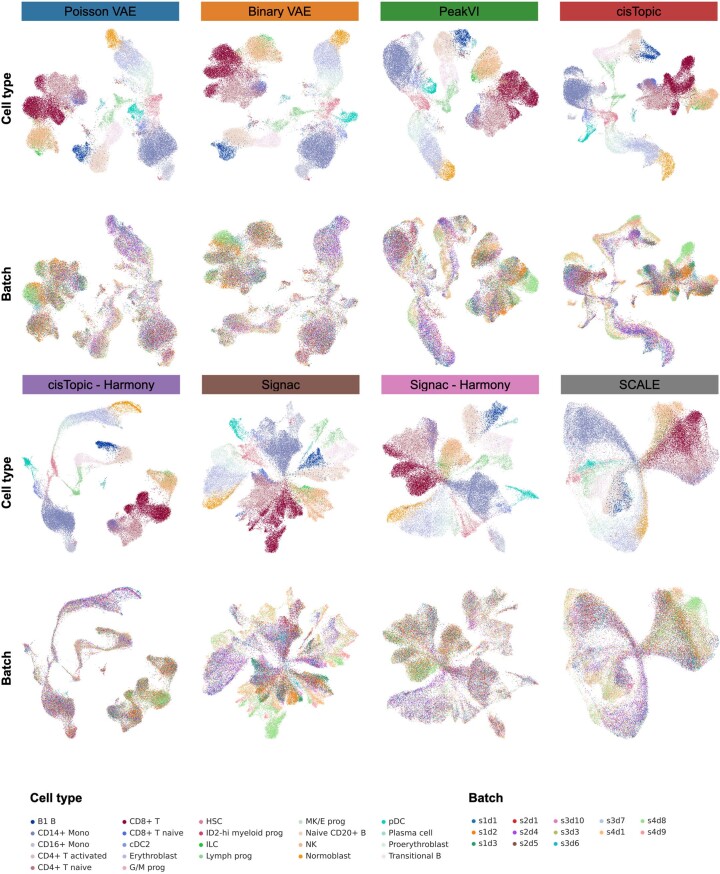
UMAPs of integrated latent space for the NeurIPS dataset. UMAP of the integrated latent space of the NeurIPS dataset using the Poisson VAE, Binary VAE, PeakVI, Signac using LSI, cisTopic using LDA, and SCALE model. Cells are colored by cell type (top row) and batch (bottom row). For cisTopic and Signac, additional batch correction was performed using Harmony.

**Extended Data Fig. 6:**
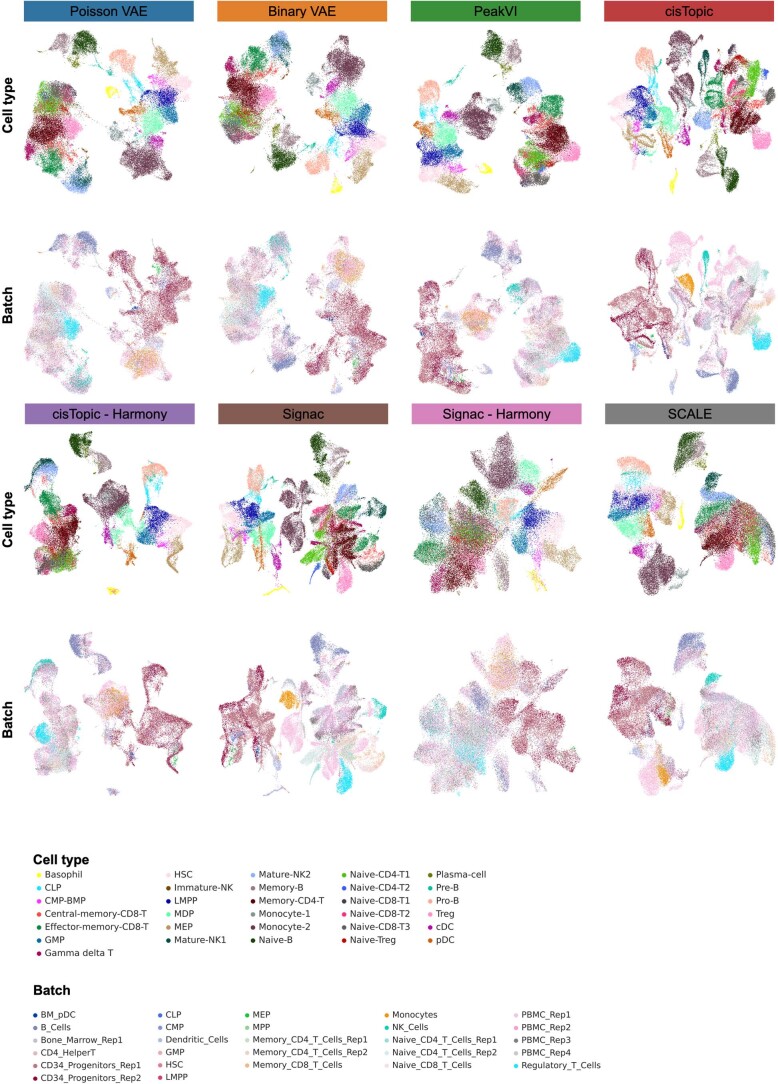
UMAPs of integrated latent space for the Satpathy dataset. UMAP of the integrated latent space of the Satpathy dataset using the Poisson VAE, Binary VAE, PeakVI, Signac using LSI, cisTopic using LDA, and SCALE model. Cells are colored by cell type (top row) and batch (bottom row). For cisTopic and Signac, additional batch correction was performed using Harmony.

**Extended Data Fig. 7:**
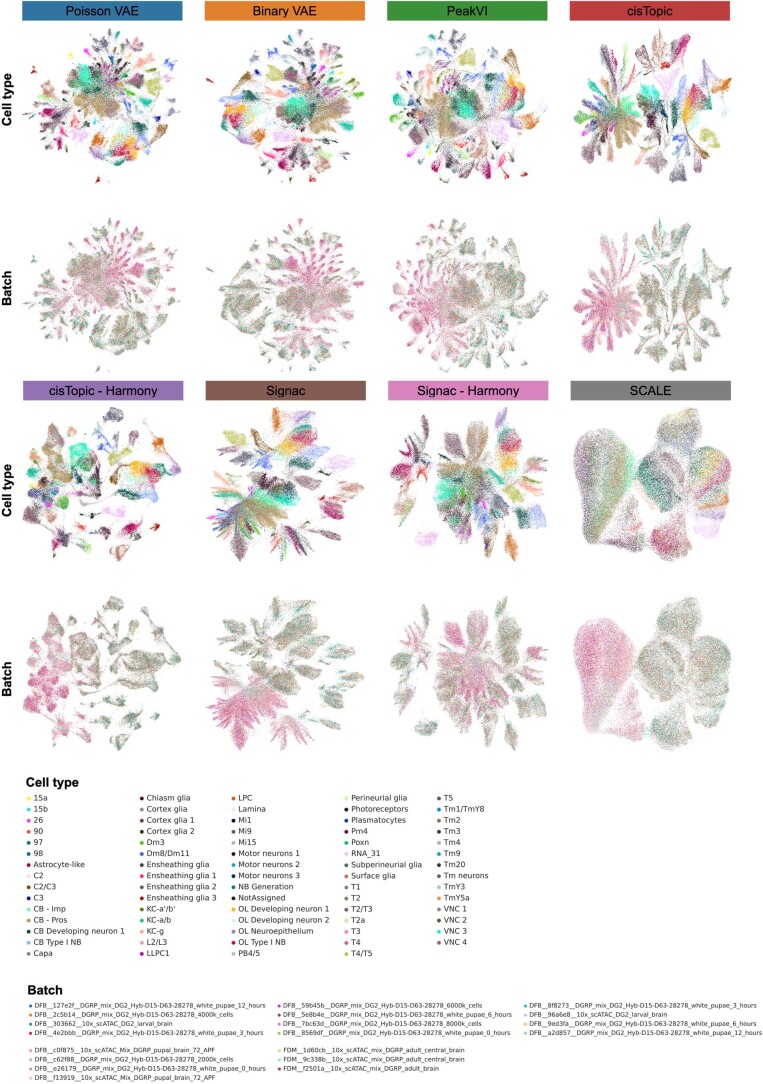
UMAPs of integrated latent space for the Fly dataset. UMAP of the integrated latent space of the fly dataset using the Poisson VAE, Binary VAE, PeakVI, Signac using LSI, isTopic using LDA, and SCALE model. Cells are colored by cell type (top row) and batch (bottom row). For cisTopic and Signac, additional batch correction was performed using Harmony.

**Extended Data Fig. 8:**
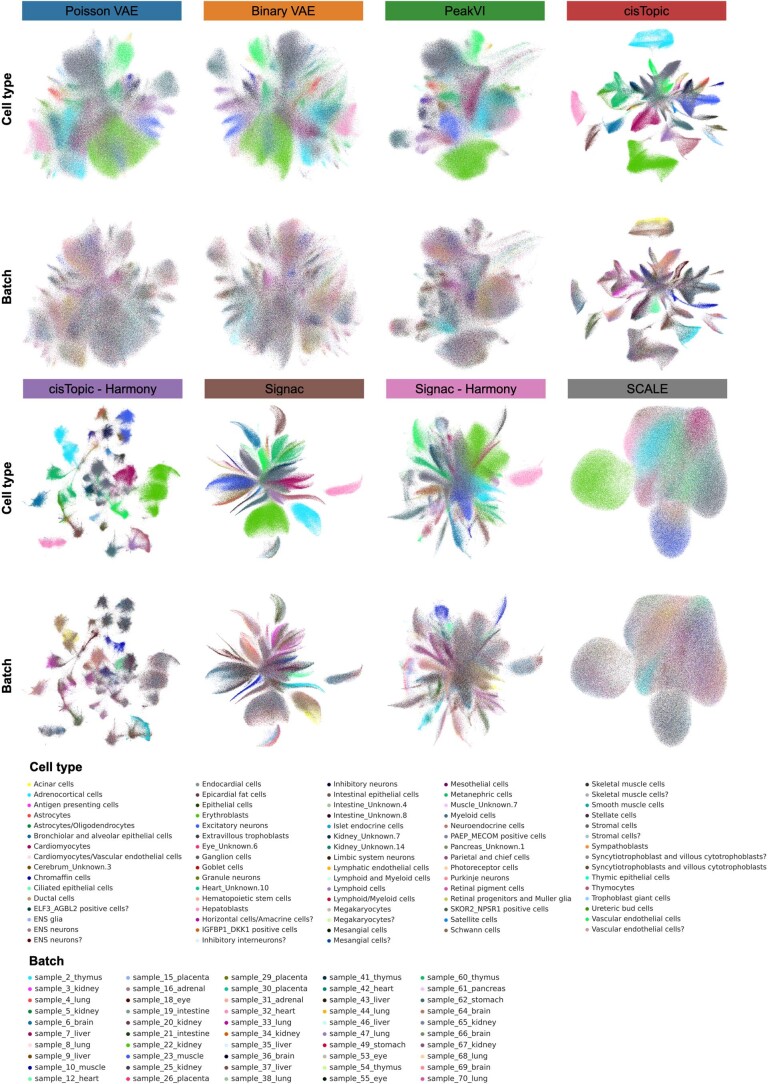
UMAPs of integrated latent space for the sci-ATAC-seq3 dataset. UMAP of the integrated latent space of the sciATAC-seq3 dataset using the Poisson VAE, Binary VAE, PeakVI, Signac using LSI, cisTopic using LDA, and SCALE model. Cells are colored by cell type (top row) and batch (bottom row). For cisTopic and Signac, additional batch correction was performed using Harmony.

We also evaluated the learnt latent representations using several integration metrics divided into two categories, batch integration and bioconservation^18^. In addition to the three VAE models, we compared the embedding techniques of three widely used methods (Supplementary Table 1): latent semantic indexing (LSI; Signac^3^ and ArchR^5^); latent Dirichlet allocation (cisTopic^8^) and SCALE^10^, a deep generative model. While binary methods performed reasonably well across the datasets, there was no apparent benefit in utilizing binarized data (Extended Data Figs. 3, 4a and 5–8). cisTopic, Signac and SCALE are not explicitly designed for batch correction and may consequently exhibit lower scores in batch correction metrics (Supplementary Table 1). Batch correction can matter, as demonstrated by the successful integration of the Kenyon cell subtype (KC-g) in the Fly dataset (Extended Data Fig. 7) achieved by Poisson VAE, Binary VAE and PeakVI, which explicitly account for batch effects. Nevertheless, our observation that binarization offered no clear benefit remained consistent across different weightings of bioconservation and batch correction metrics (Extended Data Fig. 4b).

Beyond the lack of advantage in using binarized data, preserving quantitative information can enhance cell representation. For instance, Poisson VAE better recovered the rare cell type ID2-hi myeloid progenitors in the NeurIPS dataset (Supplementary Table 1), as indicated by the improved isolated label F1 score (Fig. 2b and Extended Data Figs. 3 and 5).

**Extended Data Fig. 9:**
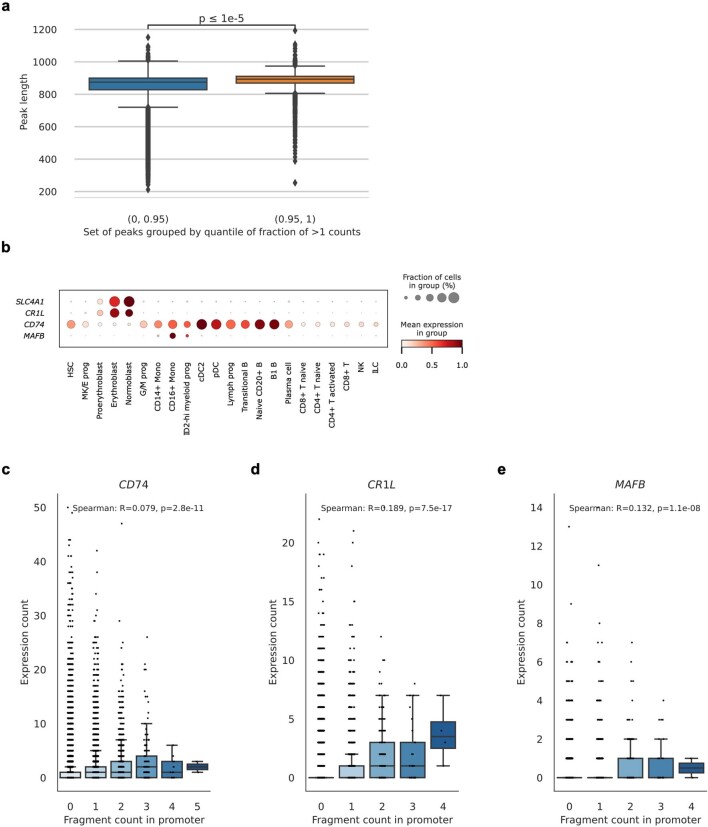
Peak length distribution and correlation of gene expression with chromatin accessibility counts for selected marker genes. **a)** Peak distribution length for peaks in the top 0.05 quantile (n = 5727) and bottom 0–0.95 quantile (n = 110,760) according to the fraction of counts above the binarization threshold. High-count peaks are significantly longer. The *P* value was computed using a two-sided Wilcoxon test. **b)** Expression of genes (rows) associated with each cell type (columns). *CR1L* is involved in the red blood cell lineage^34^ (Proerythroblast, Erythroblast, Normoblast). *CD74* is expressed in antigen-presenting cells and is known to regulate mature B-cell survival^35^. *MAFB* is a transcription factor that represses erythrocyte programs in myeloid cells^36^. Correlation of gene expression and fragment counts in the promoter of the (**c**) *CD74* gene (n = 7000), (**d**) *CR1L* gene (n = 1917), and (**e**) *MAFB* gene (n = 1845). The two-sided Spearman correlation analysis was computed on fragment counts greater than 0. *P* values were adjusted for multiple testing using the Benjamini–Hochberg correction. We restricted the plot to cells of similar total fragment count (0.25–0.75 quantile) to avoid capturing effects driven by total fragment count. We see a quantitative signal in promoter accessibility that would be lost by binarization. In all boxplots, the central line denotes the median, boxes represent the interquartile range (IQR), and whiskers show the distribution except for outliers. Outliers are all points outside 1.5 times the IQR.

**Extended Data Fig. 10:**
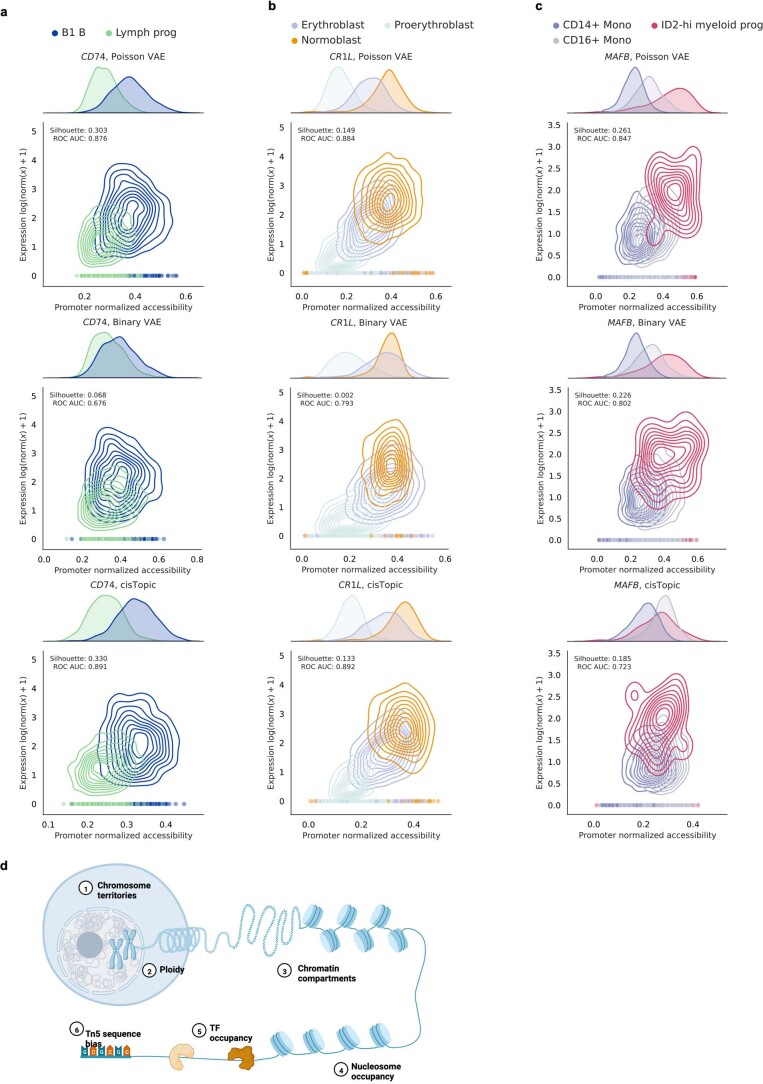
Cell type separation on promoters of marker genes. **a, b, c)** Log-normalized gene expression against normalized accessibility for the Poisson VAE (top row), Binary VAE model (middle row), and cisTopic model (bottom row) for the (**a**) *CD74* gene, (**b**) *CR1L* gene, and (**c**) *MAFB* gene. Cell type separation is measured with the silhouette width and area under the ROC curve and is better with the Poisson VAE model for *CR1L* and *MAFB* and second for *CD74*. **d)** Multiple biological factors contribute to DNA accessibility in single cells to be quantitative rather than binary. They include a diploid genome, density of chromatin packaging, nucleosome spacing, TFs in a peak region preventing the Tn5 from binding, and sequence preferences of Tn5.

We further investigated the biological signal represented by quantitative data to understand effects that could be captured in the Poisson VAE. We first examined high-count peaks and found they tend to be broader (Extended Data Fig. 9a) and enriched for promoter regions of highly expressed genes, highly variable genes and super-enhancers (Fig. 2c; Methods). Conversely, low-count peaks were associated with distal enhancer elements, consistent with previous bulk observations highlighting the accessibility differences between active transcription start sites (TSSs) and enhancers^2^. Next, we examined whether increased TSS accessibility correlated with higher gene expression using the NeurIPS dataset, focusing on cells with at least one fragment in the promoter region. We observed a significant correlation (i.e., Spearman correlation *P* < 0.05) between promoter accessibility and gene expression in 481 out of 3,879 genes (12.4%, 2.5-times higher than expected, binomial test *P* < 0.05), in agreement with a recent preprint^19^. To illustrate, we considered cell type markers among the top 20 highest correlated genes (Extended Data Fig. 9b), including *SLC4A1*, a gene involved in the red blood cell lineage^20^ (Spearman correlation 0.12, *P* = 0.001; Fig. 2b,d). Similarly, we found a significant correlation for genes involved in other biological lineages (Extended Data Fig. 9c–e). We tested whether the Poisson VAE model can capture this quantitative accessibility signal and enhance cell type discrimination in these promoter regions. Indeed, the normalized accessibility from Poisson VAE showed improved cell type separation compared to cisTopic and Binary VAE in three out of four cases (Fig. 2e–g and Extended Data Fig. 10; Methods).

In conclusion, we found that scATAC-seq binarization is unnecessary and results in a loss of useful information. What makes scATAC-seq quantitative? Chromatin accessibility is highly dynamic and nucleosome turnover rates are in the same order of magnitude as the scATAC-seq incubation duration^1,21^. Furthermore, transcription factors, not unlike transposase, must diffuse through the nucleus to access DNA, potentially reaching distinct chromosome territories and compartments with various efficiencies (Extended Data Fig. 10d). Also, a single genomic position in diploid cells may not be simultaneously open or closed on both alleles. Our observations indicate that scATAC-seq fragment counts capture this continuum of chromatin accessibility^19^. Even though the advantage of quantitative modeling is diminished for very sparse datasets, treating scATAC-seq data quantitatively is more general than binarization and it matters to study highly expressed and highly variable genes, including important marker genes. These findings have immediate practical implications as using a Poisson over a binary loss has no increase in computational cost. Future directions include investigating other typically binarized settings, such as scChIP-seq^22^ and alternative count distributions such as negative binomial.

## Methods

### Input data and preprocessing

#### NeurIPS dataset

The multiome hematopoiesis dataset from the NeurIPS 2021 challenge^13^ was downloaded from the AWS bucket s3://openproblems-bio/public/. We did not perform any additional filtering of the data. scATAC-seq BAM files were downloaded from the Gene Expression Omnibus (GEO) under accession code GSE194122.

#### Satpathy dataset

The second hematopoiesis dataset^14^ was downloaded from GEO (accession code GSE129785). Specifically, the processed count matrix and metadata files: scATAC-Hematopoiesis-All.cell-barcodes.txt.gz, scATAC-Hematopoiesis-All.mtx.gz and scATAC-Hematopoiesis-All.peaks.txt.gz. We then filtered the peaks to only those that were detected in at least 1% of the cells in the sample, reducing the data from 571,400 to 134,104 peaks.

#### Fly dataset

Raw fragment files for chromatin accessibility of the fly brain^15^ were downloaded from GEO (accession code GSE163697). Additionally, peak regions, cell barcodes and cell metadata were extracted from the cisTopic object AllTimepoints_cisTopic.Rds, which was downloaded from flybrain.aertslab.org. Fragments were counted per peak region using the Signac function FeatureMatrix. We then filtered the peaks to be detected in at least 1% of all cells. Furthermore, we excluded cells labeled unknown (CellType_lvl1 equal to ‘unk’ or ‘-’).

#### sci-ATAC-seq3 dataset

Count matrices and metadata were downloaded from GEO (accession code GSE149683)^16^. Peaks were filtered to be accessible in at least 1% of all cells.

### Fragment computation

The standard 10x protocol for generating the cell-peaks matrix is to count the fragment ends (reads). To estimate fragment counts, we rounded all uneven counts to the next highest even number and halved the resulting read counts.

### Poisson VAE model

Let ${X}^{N\times P}$ be a fragment count matrix consisting of *N* cells and *P* peak regions. We model the counts $x_{cp}$ with a variational autoencoder:

$${{{\bf{z}}}}_{{c}}\sim {\rm{Normal}}\left({{f}}^{\,{{\mu }}}\left({{{\bf{x}}}}_{{c}}\right),{{f}}^{\,{{\sigma }}}\left({{{\bf{x}}}}_{{c}}\right)\right)$$

$${{{\rho }}}_{{cp}}={{g}}_{{p}}\left({{{\bf{z}}}}_{{c}},{{s}}_{{c}}\right)$$

$${w}_{{cp}}={\rm{softmax}}\left({{{\rho }}}_{{cp}}+{{r}}_{{p}}\right)$$

$${{\lambda }}_{{cp}}=\exp \left({{l}}_{{c}}\right)\cdot {{w}}_{{cp}}$$

$${{x}}_{{cp}}\sim {\rm{Poisson}}\left({{\lambda }}_{{cp}}\right)$$

The neural networks ${{f}}^{\,{{\mu }}},{{f}}^{\,{{\sigma }}}$ encode the parameters of a multivariate normal random variable from which ***z***_*c*_ is drawn. *g*_*p*_ is a neural network that maps the latent representation ***z***_*c*_ concatenated to the batch annotation *s*_*c*_ back to the dimension of peaks. *r*_*p*_ captures a region-specific bias such as the mean fragment count or peak length and is learned directly. *l*_*c*_ refers to the log-transformed total fragment counts per cell ${l}_{c}=\log ({\sum }_{{p}}{x}_{{cp}})$. *w*_*cp*_ is constrained to encode the mean distribution of *l*_*c*_ reads over all peaks by using a softmax activation in the last layer. This means that ${\sum }_{{p}}{w}_{{cp}}=1$.

### Binary VAE model

The Binary VAE model models binarized counts:

$${{y}}_{{cp}}=\left\{\begin{array}{c}0\,{\rm{if}}\,{{x}}_{{cp}}=0\\ 1\,{\rm{if}}\,{{x}}_{{cp}} > 0\end{array}\right.$$

The binarized signal was modeled as follows:

$${{{\bf{z}}}}_{{c}}\sim {\rm{Normal}}\left({{f}}^{\,{\mu }}\left({{{\bf{y}}}}_{{c}}\right),{{f}}^{\,{\sigma }}\left({{{\bf{y}}}}_{{c}}\right)\right)$$

$${{{\rho }}}_{{cp}}={{g}}_{{p}}\left({{\rm{\bf{y}}}}_{{c}},{{s}}_{{c}}\right)$$

$${{\theta}}_{{cp}}={\sigma }\left({{{\rho }}}_{{cp}}+{{r}}_{{p}}+\widetilde{{{l}}}_{{c}}\right)$$

$${{y}}_{{cp}}\sim {\rm{Ber}}\left({{\theta}}_{{cp}}\right)$$

We included the proportion of non-zeros by modeling:

$$\widetilde{{{l}}}_{{c}}={{\sigma }}^{-1}\left(\frac{1}{P}\sum _{{p}}{{y}}_{{cp}}\right)$$

Here, σ^−1^ is the logit function. This way *θ*_*cp*_ is equal to the mean accessibility of the cell for ${{{\rho }}}_{{c}{p}}={{r}}_{{p}}=0$.

### Encoder and decoder functions

The functions ${{f}}^{\,{\rm{\mu }}},{{f}}^{\,{\rm{\sigma }}}$ and the function *g*_w_ are encoder and decoder functions, respectively. To be as comparable as possible to PeakVI as implemented in scvi-tools^9,23^ (v.0.20.3), we used the same architecture. Specifically, these networks consisted of two repeated blocks of fully connected neural networks with a fixed number of hidden dimensions set to the square root of the number of input dimensions, a dropout layer, a layer-norm layer and leakyReLU activation. The last layer in the encoder maps to a defined number of latent dimensions *n*_latent_.

### Training procedure

We used the default PeakVI training procedure with a learning rate of 0.0001, weight decay of 0.001 and minibatch size of 128 and used early stopping on the validation reconstruction loss. We used a random training, validation and test set of 80%, 10% and 10%, respectively. This was repeated ten times. We computed all evaluation metrics on the left-out test cells.

### Hyperparameter optimization

All models were run using the default PeakVI parameters. For the reconstruction task, we optimized the number of latent dimensions *n*_latent_ on the validation set for each dataset and model on reconstructing the binary accessibility matrix as measured by average precision. The used range was from 10 to 100 in increments of 10.

### Benchmarking methods

#### cisTopic

We used the Python implementation of cisTopic, pycisTopic^8,24^ (v.1.0.3.dev2+g45b7e66.d20230426). cisTopic objects were created from the binarized count matrices. We then modeled the topics using the Mallet algorithm on 10 to 100 topics in steps of 10. We selected the optimal topic number using the suggested model selection metrics Minmo_2011^25^ and log-likelihood^26^. Finally, dimensionality reduction was performed on the cell-topic matrix with optionally first running Harmony^27^ (harmonypy, v.0.0.9) to reduce batch effects.

#### SCALE

We used the provided Python script on github.com/jsxlei/SCALE to run SCALE^10^ on the binarized count matrix. We set the number of clusters to the number of cell types in the dataset.

For visualization, a two-dimensional UMAP^28^ (umap-learn, v.0.5.3) of the integrated latent space was generated based on the 15-nearest-neighbor graph. The cross-validation run with the best reconstruction was used.

#### Signac

Count matrices were loaded into ChromatinAssays using Signac^3^ (v.1.9.0) and Seurat^29^ (v.4.3.0) without additional filtering (min.cells = min.features = 0). We then computed the LSI embedding using the default procedure (RunTFIDF followed by RunSVD). We removed components that correlated with the total fragment count by more than 0.5. To investigate the effect of batch normalization, we created a batch-normalized LSI embedding by running RunHarmony with the respective batch variable as input.

### Evaluation

#### Reconstruction metrics

The reconstruction metrics were calculated on the binarized matrix. Poisson rate parameters *λ*_*cp*_ were transformed to a Bernoulli probability *θ*_*cp*_ by computing the probability of getting one or more fragments in a peak for a given cell:

$${{\theta}}_{{cp}}={\mathbb{P}}\left({{x}}_{{cp}} > 0 \mid {{\lambda}}_{{cp}}\right)=1-{\mathbb{P}}\left({{x}}_{{cp}}=0\mid{{\lambda}}_{{cp}}\right)=1-{{\rm{e}}}^{-{{\lambda}}_{{cp}}}$$

##### Average precision

As our reconstruction task is highly imbalanced (only a small fraction of all peaks are accessible), we used the average precision score as implemented in scikit-learn (v.1.2.2) to evaluate the reconstruction. Average precision estimates the area under the precision-recall curve.

#### Integration metrics

We used the scib^18^ (v.1.1.3) implementation for computing the integration metrics on the latent embedding of the cells. We used all available metrics using default parameters but excluded metrics that were specifically developed for single-cell RNA sequencing datasets (highly variable genes score and cell cycle score) and kBET due to its long run time. The trajectory score was only run for the NeurIPS dataset, which had a precomputed ATAC trajectory. Scib categorizes the metrics into metrics that measure batch correction and biology conservation.

Bioconservation comprises the following metrics that are applied to predefined cell-type labels that each dataset provided:

##### Normalized mutual information

This measures the consistency of two clusterings. Here, we compare how well a clustering on the integrated embedding agrees with predefined cell-type labels. For optimal clustering, the scib package runs Louvain clustering at resolutions ranging from 0.1–2 in steps of 0.1.

##### Adjusted Rand index

This is a different metric to compare the clusterings with the predefined cell-type labels.

##### Label silhouette width

This measures the within-cluster distance of cells compared to the distance to the closest neighboring cluster. A value close to 1 indicates a high separation between clusters. We used the predefined cell labels to define clusters for the label silhouette width calculation.

##### Graph cLISI

This measures the separation of the *k*NN graph. It evaluates the likelihood of observing the same cell-type label in the nearest neighbors, indicating good cell-type separation.

##### Isolated label metrics

The isolated labels are defined as the cell types present in the fewest number of batches (Supplementary Table 1). Two metrics evaluate how well isolated labels separate from other cell types. The F1 score is the harmonic mean of precision and recall. The isolated label silhouette measures the average silhouette width (ASW) of the isolated label compared to all non-isolated labels.

##### Trajectory conservation

This computes the correlation of inferred pseudotime ordering before and after integration.

Four metrics measure different levels of batch integration:

##### Principal component regression

This measures the amount of variance of the principal components of the embedded space that can be explained by the batch variables before and after integration.

##### Graph connectivity

This measures whether the *k*NN graph of the embedding connects all cells that have the same cell-type label. If there are strong batch effects, this will not be the case.

##### Graph iLISI

This measures the mixture of the *k*NN graph. It evaluates the likelihood of observing different batch labels in the nearest neighbors, indicating a good batch mixing.

##### Batch silhouette width

This is a metric similar to the label silhouette width but applied to batch labels. To ensure that higher scores represent better mixing, the silhouette metric is subtracted from 1. The ASW is computed separately for each cell label to assess the mixing within cells of the same label. Finally, the individual ASW scores for each cell label are averaged to obtain an overall measure of batch mixing.

### Enrichment analysis

Enrichment analysis was performed with respect to four sets of regulatory elements: distal enhancers, super-enhancers, highly expressed genes and highly variable genes.

Annotations for distal enhancers in the hg38 genome assembly were downloaded from ENCODE Registry of CREs (v.3, screen.encodeproject.org)^30^. They were then subset to distal cCREs with enhancer-like signatures (dELS) and CTCF-bound cCREs with enhancer-like signatures (CTCF-bound, dELS).

Super-enhancers were downloaded from SEdb 2.0 (www.licpathway.net/sedb/)^31^. Only bone marrow samples were included.

Highly expressed genes were computed using the preprocessed single-cell RNA sequencing data from the NeurIPS dataset. They were defined as the top 2,000 genes ranked by mean expression across all cells.

Highly variable genes were computed with scanpy^32^ (v.1.9.2) using Seurat-based highly variable gene selection with default parameter settings.

We filtered annotations to overlap with at least one peak of the NeurIPS dataset. Region overlap was determined using the pyRanges package (v.0.0.124). Odds ratios and significance were computed using the Fisher exact test implemented in scipy (v.1.10.1) and corrected for multiple testing with Benjamini–Hochberg at a false discovery rate of 0.05.

### Correlation with gene expression analysis

We used the peak annotation of CellRanger ATAC to subset high-count peaks to promoter regions. CellRanger annotates a peak as a promoter if it overlaps with the promoter region (−1,000 bp, +100 bp) of any transcription start site^4^. Then, we computed the Spearman correlation between a cell’s fragment count in the promoter peaks and the gene expression count using scipy, taking only cells with a fragment count >1 into account. As this correlation can be driven by cells with a high total fragment count, we restricted the computation to cells whose total fragment count was in the 0.25–0.75 quantile.

### Normalized accessibility

We can use the learned latent space and generative model of Poisson VAE and Binary VAE to produce denoised and normalized estimates of accessibility, controlling for sequencing depth^23^. To this end, we defined the normalized accessibility of the model output using the median total fragment count across all cells. For cisTopic, we used the imputed and normalized accessibility scores.

We compared the normalized accessibility of the models by computing the cell type separation using the silhouette width and ROC AUC.

### Reporting summary

Further information on research design is available in the Nature Portfolio Reporting Summary linked to this article.

## Online content

Any methods, additional references, Nature Portfolio reporting summaries, source data, extended data, supplementary information, acknowledgements, peer review information; details of author contributions and competing interests; and statements of data and code availability are available at 10.1038/s41592-023-02112-6.

### Supplementary information

Reporting Summary

Supplementary Table 1 Description of the datasets and detailed information on scATAC-seq methods including their counting and binarization strategy.

## Data Availability

Raw published data for the NeurIPS, Satpathy, the Fly and the sci-ATAC-seq3 datasets are available from the GEO under accession codes GSE194122, GSE129785, GSE163697 and GSE149683, respectively. Annotations for distal enhancers in the hg38 genome assembly were downloaded from ENCODE Registry of CREs (v.3, screen.encodeproject.org). Super-enhancers were downloaded from SEdb v.2.0 (www.licpathway.net/sedb/).

## Code availability

All models, code and notebooks to reproduce our analysis and figures, as well as a tutorial notebook to use the Poisson VAE model, are available at github.com/theislab/scatac_poisson_reproducibility. The code has additionally been archived and is available on Zenodo at 10.5281/zenodo.8356171 (ref. ^33^). The Poisson VAE model is available as an extension of the scvi-tools suite at github.com/lauradmartens/scvi-tools.
