# Supplementary material for: Modeling fragment counts improves single-cell ATAC-seq analysis
Source: Nat Methods. 2023 Dec 4;21(1):28–31. doi: 10.1038/s41592-023-02112-6 (PMC10776385; doi:10.1038/s41592-023-02112-6)
Supplement: Supplementary file 1 — Reporting Summary [file 41592_2023_2112_MOESM1_ESM.pdf]

nature portfolio

Corresponding author(s): Julien Gagneur
Fabian Theis

Last updated by author(s): 15 September 2023

Reporting Summary

Nature Portfolio wishes to improve the reproducibility of the work that we publish. This form provides structure for consistency and transparency in reporting. For further information on Nature Portfolio policies, see our Editorial Policies and the Editorial Policy Checklist.

Statistics

For all statistical analyses, confirm that the following items are present in the figure legend, table legend, main text, or Methods section.

n/a	Confirmed	
☐	☒	The exact sample size (*n*) for each experimental group/condition, given as a discrete number and unit of measurement
☒	☐	A statement on whether measurements were taken from distinct samples or whether the same sample was measured repeatedly
☐	☒	The statistical test(s) used AND whether they are one- or two-sided<br>*Only common tests should be described solely by name; describe more complex techniques in the Methods section.*
☒	☐	A description of all covariates tested
☐	☒	A description of any assumptions or corrections, such as tests of normality and adjustment for multiple comparisons
☐	☒	A full description of the statistical parameters including central tendency (e.g. means) or other basic estimates (e.g. regression coefficient) AND variation (e.g. standard deviation) or associated estimates of uncertainty (e.g. confidence intervals)
☐	☒	For null hypothesis testing, the test statistic (e.g. *F*, *t*, *r*) with confidence intervals, effect sizes, degrees of freedom and *P* value noted<br>*Give P values as exact values whenever suitable.*
☒	☐	For Bayesian analysis, information on the choice of priors and Markov chain Monte Carlo settings
☒	☐	For hierarchical and complex designs, identification of the appropriate level for tests and full reporting of outcomes
☐	☒	Estimates of effect sizes (e.g. Cohen's *d*, Pearson's *r*), indicating how they were calculated

Our web collection on statistics for biologists contains articles on many of the points above.

Software and code

Policy information about availability of computer code

Data collection	No software was used to collect data.
Data analysis	We used the following python packages: scanpy (v.1.9.2), pycisTopic (v.1.0.3.dev2+g45b7e66.d20230426), harmonypy, v.0.0.9, scvi-tools (v.0.20.3), scib (v.1.1.3), scikit-learn (v.1.2.2), pyRanges (v.0.0.124), scipy (v.1.10.1), umap-learn (v.0.5.3) and the following R packages: Signac (v.1.9.0), Seurat (v.4.3.0). For SCALE we used the provided Python script on https://github.com/jsxlei/SCALE.<br>All models, code, and notebooks to reproduce our analysis and figures are available on https://github.com/theislab/scatac_poisson_reproducibility including documentation and examples.

For manuscripts utilizing custom algorithms or software that are central to the research but not yet described in published literature, software must be made available to editors and reviewers. We strongly encourage code deposition in a community repository (e.g. GitHub). See the Nature Portfolio guidelines for submitting code & software for further information.

Data

Policy information about availability of data

All manuscripts must include a data availability statement. This statement should provide the following information, where applicable:

- Accession codes, unique identifiers, or web links for publicly available datasets
- A description of any restrictions on data availability
- For clinical datasets or third party data, please ensure that the statement adheres to our policy

Raw published data for the NeurIPS, Satpathy, the fly, and the sci-ATAC-seq3 datasets are available from the Gene Expression Omnibus under accession codes GSE194122, GSE129785, GSE163697, and GSE149683, respectively. Annotations for distal enhancers in the hg38 genome assembly were downloaded from ENCODE Registry of CREs (v3, screen.encodeproject.org). Super-enhancers were downloaded from SEdb 2.0 (http://www.licpathway.net/sedb/).

Field-specific reporting

Please select the one below that is the best fit for your research. If you are not sure, read the appropriate sections before making your selection.

☒ Life sciences ☐ Behavioural & social sciences ☐ Ecological, evolutionary & environmental sciences

For a reference copy of the document with all sections, see nature.com/documents/nr-reporting-summary-flat.pdf

Life sciences study design

All studies must disclose on these points even when the disclosure is negative.

Sample size	This study uses only published datasets from prior studies. We chose the four representative datasets to represent different organisms, tissues and protocols.
Data exclusions	Peaks from the datasets were excluded when they had counts in less than 1% of the cells. Some cells were excluded if they did not have a meaningful cell type annotation (e.g. Unkown).
Replication	We developed computational methods and evaluated and benchmarked these methods on a diverse set of datasets. For each tested model we ran 10 cross-validations on subsets of the data to evaluate the robustness of the results.
Randomization	Randomization was not performed. Statistical test were performed on matched data subsets where no confounding by additional factors was expected.
Blinding	Method evaluation was performed on each of the four datasets using unbiased metrics (e.g. reconstruction in average precision). Data analysis was not blinded as we required metadata availability (cell types, batch).

Reporting for specific materials, systems and methods

We require information from authors about some types of materials, experimental systems and methods used in many studies. Here, indicate whether each material, system or method listed is relevant to your study. If you are not sure if a list item applies to your research, read the appropriate section before selecting a response.

Materials & experimental systems

n/a	Involved in the study
☒	☐ Antibodies
☒	☐ Eukaryotic cell lines
☒	☐ Palaeontology and archaeology
☒	☐ Animals and other organisms
☒	☐ Human research participants
☒	☐ Clinical data
☒	☐ Dual use research of concern

Methods

n/a	Involved in the study
☒	☐ ChIP-seq
☒	☐ Flow cytometry
☒	☐ MRI-based neuroimaging

March 2021
